# pH-Responsive Amphiphilic Carboxylate Polymers: Design and Potential for Endosomal Escape

**DOI:** 10.3389/fchem.2021.645297

**Published:** 2021-03-23

**Authors:** Shiqi Wang

**Affiliations:** Drug Research Program, Division of Pharmaceutical Chemistry and Technology, Faculty of Pharmacy, University of Helsinki, Helsinki, Finland

**Keywords:** pH, intracellular delivery, amphiphilicity, polymeric materials, drug delivery and targeting

## Abstract

The intracellular delivery of emerging biomacromolecular therapeutics, such as genes, peptides, and proteins, remains a great challenge. Unlike small hydrophobic drugs, these biotherapeutics are impermeable to the cell membrane, thus relying on the endocytic pathways for cell entry. After endocytosis, they are entrapped in the endosomes and finally degraded in lysosomes. To overcome these barriers, many carriers have been developed to facilitate the endosomal escape of these biomacromolecules. This mini-review focuses on the development of anionic pH-responsive amphiphilic carboxylate polymers for endosomal escape applications, including the design and synthesis of these polymers, the mechanistic insights of their endosomal escape capability, the challenges in the field, and future opportunities.

## Introduction

Most successfully developed biotherapeutics up to date only target extracellular receptors, because the intracellular delivery of biomacromolecules remains a key challenge (Stewart et al., 2018; van Haasteren et al., 2020). Such challenge resides in the natural barrier of plasma membranes, composed of a lipid bilayer and membrane proteins. The permeability of the plasma membrane is specifically selective. Therefore, biomacromolecular therapeutics, such as proteins, peptides, and genes, are blocked from free movement across the plasma membrane (Pei and Buyanova, 2019). Instead, these biomacromolecules are mostly internalized by endocytosis. After internalization, they are trapped in endosomes, and finally degraded within lysosomes. Thus, it is critical to develop carriers to facilitate the endosomal escape and release the payloads in cytoplasm, to maximize their therapeutic potential.

Polymer carriers for endosomal escape purposes have been developed for years. Specifically, pH-responsive polymers have attracted significant attention (Cupic et al., 2019; Deirram et al., 2019), because their endosomal escape property is activated by the pH differences between the extracellular physiological environment (7.4) and the acidic endosomal environment (6.0–6.8 in early endosomes, 5.0–6.0 in late endosomes, and 4.5–5.0 in lysosomes)(Mukherjee et al., 1997; Scott et al., 2014). According to the ionizable groups, there are two main pH-responsive polymer categories: polycations and polyanions (Bazban-Shotorbani et al., 2017). Polycations have weak basic functional groups, such as amines, imidazole, and pyridine, which become positively charged when the pH drop below their pK_a_. These polymers [i.e., polyethylenimine, poly(_L_-lysine), poly(amino ester), poly(2-(dimethylamino)ethyl methacrylate), and polyamidoamine] can buffer the endosomal acidification by protonation, and cause osmotic swelling of endosomes, finally leading to endosomal rupture. The polycation induced “proton sponge” effect has long been studied, and reviewed recently (Bus et al., 2018; Vermeulen et al., 2018). In addition to the endosomal escape properties, polycations condensate genes by electrostatic interactions effectively, and thus are widely used for gene therapy (Chen et al., 2019).

**Figure 1:**
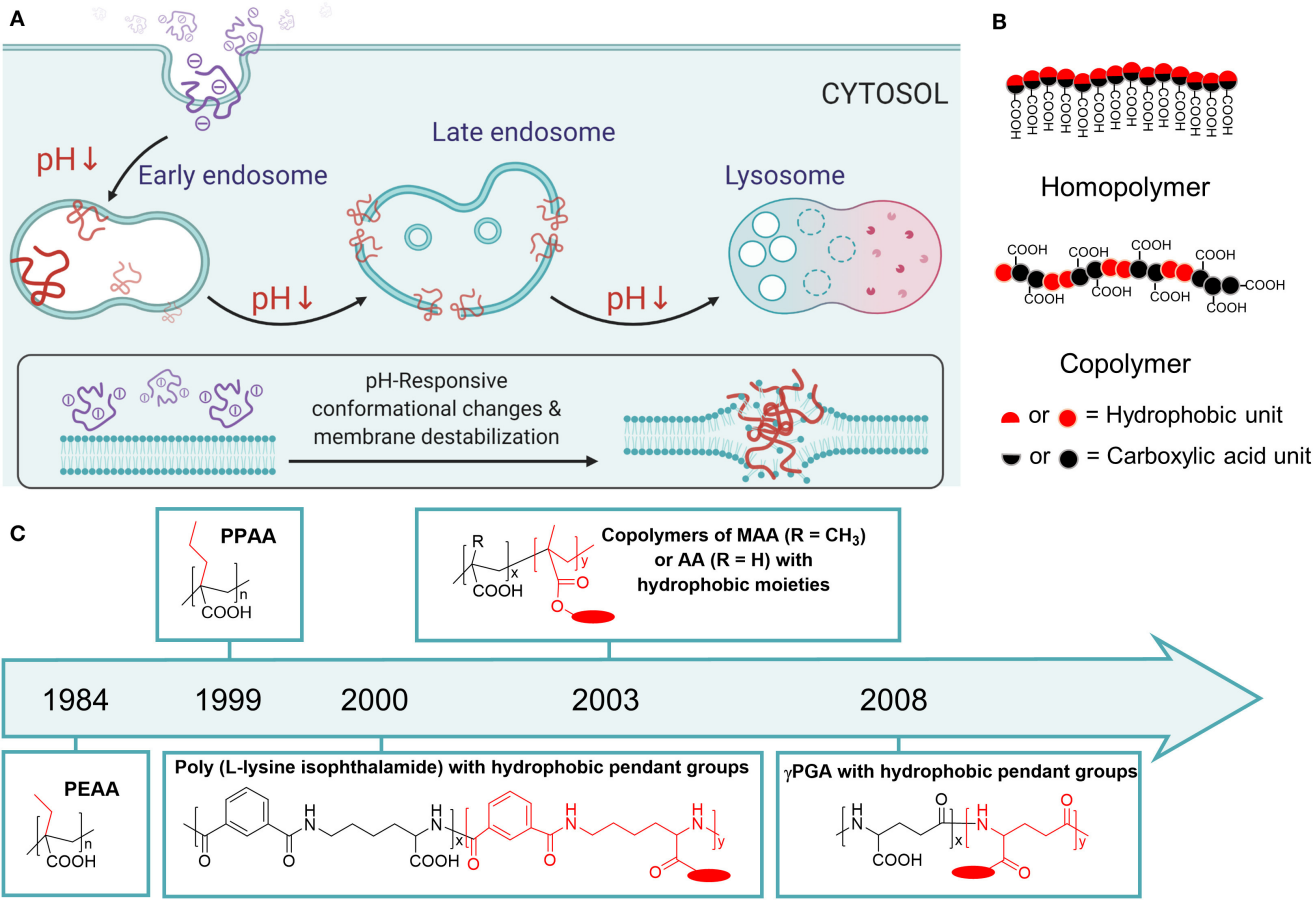
**(A)** The scheme of pH-responsive membrane permeabilizing polymers and how they facilitate endosomal escape. Created by Biorender.com **(B)** The general design of carboxylated polyanions for endosomal escape applications. **(C)** Chronological development of carboxylated polymers for endosomal escape applications. The time plotted indicated the first application for pH-dependent membrane permeabilization, instead of the first reported synthesis of the polymer.

Polyanions use a different strategy for endosomal escape. Typically, these polymers are negatively charged at physiological pH. The negative charges make them repulsive to the negatively charged plasma membranes and show non-membrane lytic property (Figure 1A). However, when pH drops below their pK_a_ in the endosomes, they lose the charge and become hydrophobic. The pH-induced alterations in the overall charges, amphiphilicity, and conformation lead to enhanced interaction with endosomal membranes and finally cause membrane disruption to release the payload into the cytoplasm (Figure 1A). Compared with polycationic polymeric carriers, anionic pH-responsive membrane permeabilizing polymers are less toxic, because of the repulsive charges against plasma membranes (Wang, 2018; Evans et al., 2019). Albeit less renowned, recent studies show their emerging potentials for proteins, genes, and vaccine delivery (Mukalel et al., 2018; Qiu et al., 2018; Evans et al., 2019; Jacobson et al., 2019; Kopytynski et al., 2020).

Herein, this mini-review introduces the development of polyanions with carboxylic acid pendant groups for endosomal escape applications. These polymers usually have two units to fulfill their function, carboxylic acid unit and hydrophobic unit (Figure 1B). The carboxylic acid unit is for pH-responsiveness, and the hydrophobic unit is for enhancing the interaction with lipid membranes. These two units could be integrated in one monomer (homopolymer), or distributed on two different monomers (copolymer) (Figure 1B). Notable examples of homopolymers include poly(ethylacrylic acid) (PEAA) and poly(propylacrylic acid) (PPAA) (Figure 1C), which have been developed for two decades for gene and protein delivery. Copolymers could be developed by copolymerization of methacrylic acid (MAA) or acrylic acid (AA) as the carboxylic acid unit, and hydrophobic methacrylates (Figure 1C). Alternatively, copolymers with different backbones, such as polypeptides and pseudopeptides, could be developed by post-polymerization modification of hydrophobic units (Figure 1C). In the following sections, the endosomal escape capability of these polymers and their functionalized derivatives in drug delivery applications are analyzed. Finally, the current challenges of pH-responsive endosomal escape polyanions development are discussed, as well as the future opportunities to be exploited.

## Amphiphilic Carboxylate Polymers: History and Recent Development

### PEAA, PPAA, and Their Derivatives

Amphiphilic carboxylate polymers were first found to have pH-dependent membrane permeabilizing capability on liposomal membranes made of egg phosphatidylcholine (PC) lipids in the 1980s (Seki and Tirrell, 1984). This effect was initially studied using PEAA, which showed significant membrane disruptive activity at its pK_a_ (6.5), without disrupting the membrane at physiological pH (Thomas and Tirrell, 1992). The membrane disruption was attributed to the pH-dependent coil-to-globule conformational transition, evidenced by hydrodynamic size variation and the desolvation of a hydrophobic fluorescent probe pyrene (Eum et al., 1989). The uncharged polymer with globule conformation could associate with the lipid membrane and even lysed the membrane completely at high polymer/lipid ratios (Thomas et al., 1994). Although these early studies based on PEAA interaction with liposomes did not explore the pH-dependent membrane permeabilizing effects on mammalian cells for intracellular delivery purposes, such findings and the coil-to-globule conformational transition mechanism laid the foundation of membrane permeabilizing polyanions. Furthermore, the research methods used by Tirrell et al. (such as pyrene fluorescent probes, interactions with model liposomal membranes, etc.) to evaluate the polymer conformation, critical pH, and to quantify membrane permeability, have been widely adopted in the following studies within the field.

PPAA (or PPAAc in some literature) has been developed in the late 1990s (Murthy et al., 1999). Compared with PEAA, PPAA has a slightly longer pendant alkyl group on the monomer, which makes it more hydrophobic (Figure 1C). Murthy et al. used red blood cells, instead of simple liposomes, to evaluate the pH-dependent membrane permeability of PPAA. Compared with liposomal membrane models, red blood cell membranes are more complicated, composed of not only lipids but also proteins and polysaccharides. The hemolytic activity, thus, was considered to better reflect the permeabilization capability toward biological membranes (Evans et al., 2013). PPAA showed higher hemolytic activity at acidic pH than PEAA without hemolytic activity at physiological pH at equivalent concentrations. It was speculated that PPAA could form pores on red blood cell membranes only at acidic pH which caused hemolysis (Murthy et al., 1999).

PPAA has been explored on mammalian cells, to enhance the gene transfection efficiency and to enhance the stability of cationic lipid gene vectors in serum (Cheung et al., 2001). The conjugation of PPAA on proteins, peptides, or antibodies by biotin-streptavidin ligation facilitated the intracellular delivery of these macromolecular cargos into the cytoplasm (Lackey et al., 2002; Albarran et al., 2011; Berguig et al., 2012). Other than chemical ligation, PPAA could form nano-polyplex by simply mixing with positively charged peptide cargos in PBS buffer (Evans et al., 2015a; Qiu et al., 2018). This approach was applicable to larger cationic cargos, including nucleic acids, gene editing ribonucleoproteins, and even nanoparticles (Evans et al., 2019). PPAA could also be formulated as polymer blends with poly(lactic-co-glycolic acid) (PLGA), to deliver antigens for T cell activation (Yang et al., 2017; Fernando et al., 2018). A detailed summary of PPAA related bioapplications is listed in Table 1.

**Table 1:** Bioapplications of PPAA and its derivatives.

**Polymer**	**Therapeutic payloads**	**Loading method**	**Bioapplications**	**References**
PPAA	DNA plasmids, antisense DNA	Mixture with cationic lipids via electrostatic interaction	Gene transfection *in vitro* and *in vivo*	Cheung et al., 2001; Kyriakides et al., 2002; Jones et al., 2003; Lee et al., 2006
PPAA	Antibodies and peptides	Chemical ligation via biotin-streptavidin	Protein and peptide intracellular delivery *in vitro*	Lackey et al., 2002; Albarran et al., 2011; Berguig et al., 2012
PPAA	Cationic MAPKAP kinase 2 inhibitor peptide	Electrostatic interaction	Inhibit pathological vasoconstriction *in vitro* and *in vivo*	Evans et al., 2015a,b
PPAA	Peptide antigens with oligolysine tails	Electrostatic interaction	Induce cellular immunity as cancer vaccines *in vivo*	Qiu et al., 2018
PPAA	Ovalbumin	Blend with PLGA, double emulsion	Induce cellular immunity *in vitro*	Yang et al., 2017
PPAA	Cationic peptides, recombinant proteins, morpholinos, and nanoparticles	Electrostatic interaction	Intracellular delivery *in vitro*	Evans et al., 2019
Co-polymer of PAA, BMA, DMAEMA	siRNA	Electrostatic interaction	Gene knockdown *in vitro*	Convertine et al., 2009, 2010; Palanca-Wessels et al., 2011
Co-polymer of PAA, BMA, DMAEMA	Ovalbumin	Blend with PLGA, double emulsion	Induce humoral and cellular immunity *in vivo*	Tran et al., 2014; Zhan and Shen, 2015
Co-polymer of PAA, and PDSEMA	Ovalbumin	Disulfide linkage with PDSEMA	Induce humoral and cellular immunity *in vivo*	Foster et al., 2010
Co-polymer of PAA, BMA, PDSEMA, HPMA	siRNA or ovalbumin	Disulfide linkage with PDSEMA	Gene knockdown or induce cellular immunity *in vivo*	Lundy et al., 2013; Keller et al., 2014
Co-polymer of PAA, BMA, PDSEMA, DMAEMA	CpG oligonucleotide (ODN) as adjuvants and ovalbumin	CpG ODN via electrostatic interaction and ovalbumin via disulfide linkage	Induce humoral and cellular immunity *in vivo*	Wilson et al., 2013; Knight et al., 2019
PEG or Jeffamine conjungated PPAA	ODNs	Mixture with cationic lipids via electrostatic interaction	Gene knockdown *in vitro* and *in vivo*	Peddada et al., 2009, 2014

The mechanism of PPAA mediated endosomal escape is closely associated with endosomal acidification since the escape process was prone to H^+^-ATPase inhibition on the endosomal membrane (Jones et al., 2003; Evans et al., 2015a). Without endosomal acidification, the carboxylic acid groups of PPAA kept deprotonated, making the polymer negatively charged and non-lytic to endosomal membranes. This means the endosomal escape property of PPAA is dependent on the pH-induced membrane permeabilization. Further studies by real-time imaging showed the intracellular delivery was correlated with galectin-8 (Gal8) recruitment, which confirms endosomal membrane damage by PPAA (Kilchrist et al., 2016). The damaged endosomes were subsequently autophaged by a “self-repaired” mechanism to avoid cell death caused by accidental endosomolytic reagents (Skowyra et al., 2018). This Gal8-mediated endosomal autophage suggests although PPAA caused damage to endosomal membranes to release the cargos intracellularly, the damage could be repaired by cells using an existing toolset.

PPAA functional derivatives, either by co-polymerization with other monomers or by changing the polymer architecture via end-to-end chemical ligations, have been widely reported for different intracellular delivery applications (Table 1). One of the most studied PPAA derivatives is the co-polymer of propylacrylic acid (PAA), dimethylaminoethyl methacrylate (DMAEMA), and butyl methacrylate (BMA). DMAEMA has a tertiary amine group, which is cationic at physiological pH (Agarwal et al., 2012). Therefore, it allows for binding with negatively charged DNA or RNA by electrostatic interaction. BMA has a butyl pendent group, which could enhance the hydrophobicity and membrane permeabilization capability at acidic pH (El-Sayed et al., 2005). A systematic investigation of the ratio of BMA in the final polymer suggested BMA-rich polymer not only showed higher hemolytic activity at pH 5.8 but also elevated the gene delivery efficiency (Convertine et al., 2009). Further studies used DMAEMA and BMA copolymerized PAA for vaccine deliveries, by covalently conjugating antigen on a thiol-reactive pyridyl disulfide monomer (PDSEMA) (Wilson et al., 2013; Knight et al., 2019). Even without adjuvant, this carrier can promote antigen presenting on dendritic cells, and enhanced antigen-specific cytotoxic T cell responses (Keller et al., 2014).

Another common type of derivatives involves the incorporation of hydrophilic blocks in the copolymer, such as poly(N-(2-hydroxypropyl) methacrylamide) (HPMA), polyethylene glycol (PEG also named as PEO), or poly(oxyalkylene amine) (Jeffamine). The PEG block could enhance polymer solubility by forming micelles and increase the resistance to serum proteins (Peddada et al., 2014; Porfiryeva et al., 2020). However, both PEG and Jeffamine conjugated PPAA showed reduced the pH-dependent membrane-lytic activity (Peddada et al., 2009). This means the endosomal membrane disruption of these PEG and Jeffamine modified derivative polymers is less than PPAA itself. In the *in vivo* study, Jeffamine conjugated PPAA showed better overall gene delivery efficiency than PPAA, probably due to the enhanced serum stability (Peddada et al., 2014). These results suggest that selecting the polymer with the best endosomal escape capability does not always end up with the most optimal delivery performance *in vivo*. Instead, balancing the endosomal escape and serum stability in the PPAA derivative polymer is important to the delivery system.

Besides linear PPAA, hyperbranched and brush-like PPAA derivatives have been developed to study the effect of polymer architecture on pH-dependent membrane permeabilizing activity. Introducing multivinyl branching monomer poly(ethylene glycol diacrylate) in the polymerization with PAA monomer generated hyperbranched PPAA, which showed lower hemolytic activity than linear PPAA at endosomal pH conditions (Tai et al., 2012). This is probably due to the limitation of conformational changes from the branching points, which weakened the membrane interaction. Brush-like PPAA, synthesized by a “graft-to” strategy after polymerization by click chemistry, showed similar pH-dependent hemolytic activity at the same mass concentration (Crownover et al., 2011).

### Copolymers of MAA or AA With Hydrophobic Moieties

Similar to PPAA, the amphiphilic copolymers of MAA (or AA) with hydrophobic methacrylates have the coil-to-globule conformational transition, when the pH decreases from neutral to acidic ranges (Kusonwiriyawong et al., 2003; Yessine et al., 2003). These polymers have pH-responsive carboxylate pendant groups from MAA, and hydrophobicity from non-ionizable methacrylates, such as BMA, dodecyl methacrylate (DMA), lauryl methacrylate (LMA), and cholesteryl methacrylate (CMA). Previous studies found that incorporating a small portion of hydrophobic monomers (i.e., 1% DMA, 2% CMA or 10% LMA) in PMAA copolymers could enhance the interaction with lipid membranes significantly, compared with PMAA homopolymer (Cho et al., 2016; Sevimli et al., 2017; Wannasarit et al., 2019). However, a further increase of the hydrophobic moieties (i.e., 8% CMA and 40% LMA) in the copolymer led to decreased solubility and enhanced supermolecular assembly in aqueous solutions, which in turn decreased the interaction between polymer and lipid membranes (Sevimli et al., 2012; Wannasarit et al., 2019). Therefore, it is critical to find the balance between the hydrophobic and hydrophilic monomers in the copolymer, to maximize the membrane association.

Regarding the applications of these MAA or AA-containing amphiphilic copolymers, a common approach is to decorate these polymers on the surface of liposomes and boost the delivery efficiency by enhancing the endosomal escape (Yessine et al., 2006; Yamazaki et al., 2017). Due to the anionic nature of these polymers, it is difficult to condensate DNA or RNA directly, but adding a cationic polymer in the formulation such as polylysine solves the problem by forming tertiary polyplexes via electrostatic interactions (Sevimli et al., 2013). A recent study indicated that these polymers can modify cell membranes by hydrophobic interactions and facilitate the delivery of cationic peptides (Dailing et al., 2020).

### Amphiphilic Carboxylated Polypeptides and Pseudopeptides

Apart from acrylic and acrylate polymers, there are also amphiphilic carboxylated polypeptides reported for pH-responsive membrane permeabilizing applications. These polypeptides are considered to be more biocompatible and biodegradable than their vinyl polymer counterparts (Akagi et al., 2006; Liu et al., 2019). A systematically investigated example is poly(γ-glutamic acid) (γPGA) and its derivatives grafted by different amino acids as pedant groups (Shima et al., 2014a). The protonation/deprotonation of glutamic acid units of PGA enabled the pH-dependent conformation changes, while the hydrophobic amino acids (e.g., leucine, methionine, phenylalanine, valine, and tryptophan) enhanced the hydrophobicity and interaction with membranes. Unlike PPAA, γPGA with sufficient hydrophobic amino acid group grafting (53% phenylalanine, 71% tryptophan, and 87% leucine) formed stable nanoparticle in PBS buffer, and the nanoparticles maintained pH-responsive hemolytic activity similar to polymers (Akagi et al., 2010; Shima et al., 2014a). Furthermore, phenylalanine modified γPGA could encapsulate protein during its self-assembly and delivered protein payload to antigen presenting cells efficiently both *in vitro* and *in vivo* (Yoshikawa et al., 2008; Akagi et al., 2011). As a natural polymer derived from *Bacillus*, γPGA itself acted as an adjuvant for both innate and adaptive immunity activation and showed promising potentials for vaccine development (Uto et al., 2011). Interestingly, it was found that both the hemolytic activity at endosomal pH, and the activation potential of antigen presenting cells increased proportionally to the hydrophobicity of the nanoparticles (Shima et al., 2013, 2014b).

A similar series of studies, using amphiphilic synthetic pseudopeptides namely poly (_L_-lysine isophthalamide) (PLP), also confirm that pH-dependent membrane-permeabilizing capability could be adjusted by grafting amino acids with different hydrophobicity or alkyl chains (Eccleston et al., 2000; Chen et al., 2009, 2017). Increasing the hydrophobicity moieties or changing the polymer structure from linear to branched could increase the interaction with lipid membranes (Wang and Chen, 2017; Chen et al., 2020). Mechanistic insights suggest that phenylalanine modified PLP induced red blood cell membrane thinning of 35–40% normal thickness at endosomal pH, thus facilitating the transport of membrane-impermeable small molecular cargos (Lynch et al., 2011). Further real-time imaging showed that even large molecules such as FITC-labeled dextran of different molecular weights (10–500 kDa) and green fluorescence protein could be delivered to different mammalian cells after co-incubation with phenylalanine modified PLP at pH 6.5 (Kopytynski et al., 2020). Such a convenient and flexible method provides a versatile platform for cell engineering *ex vivo*.

## Discussions and Future Opportunities

Since the pioneering studies of PEAA with artificial lipid membranes, there have been almost 40 years of investigation into amphiphilic carboxylate polymers for endosomal escape applications. During these years, we have witnessed significant achievements in polymer synthesis and functionalization, which enables more control over the polymer structure. One of the biggest achievements is the development of controlled radical polymerization, especially reversible addition-fragmentation chain transfer (RAFT) polymerization (Fairbanks et al., 2015; Perrier, 2017). Because RAFT polymerization is compatible with carboxylate monomers and suitable at various conditions (such as in aqueous solutions or at ambient temperature), it has been widely adopted in the amphiphilic carboxylate polymers synthesis, including PPAA derivatives and copolymers of MAA or AA mentioned in the previous section (Convertine et al., 2009; Tai et al., 2012; Sevimli et al., 2013; Wannasarit et al., 2019; Dailing et al., 2020; Wang et al., 2020).

Meanwhile, the mechanism of polymer-mediated endosomal escape has been intensively explored, along with the advances in the basic understanding of the endocytosis process itself (Skowyra et al., 2018; Vermeulen et al., 2018; Brock et al., 2019; Patel et al., 2019; Pei and Buyanova, 2019). Molecular dynamics simulation and biophysical characterizations are commonly used to provide mechanistic insights into the interaction between polymers and artificial membranes at the molecular level (Scoppola and Schneck, 2018; Sen et al., 2018), while live imaging by fluorescence microscopy captures the endosomal escape on mammalian cells (Deprey et al., 2019). The imaging gives a direct visual presentation of the polymer and the endosomes labeled by fluorescent probes with a temporal-spatial resolution. Furthermore, incorporating different endocytosis inhibitors can help to investigate which endocytosis pathway the polymers utilize and whether endosomal acidification is required for the escape (Guo et al., 2015).

Notwithstanding the great achievements in both polymer synthesis tools and endosomal escape mechanism investigation, many fundamental issues remain to be addressed in this field. For example, the fate of the amphiphilic carboxylate polymers within the cells after the endosomal escape is rarely covered in the previous publications. It is not known whether the polymer carrier itself undergoes degradation in the cell, or gets expelled from the cell somehow. This issue is critical for biomedical applications, because of the long-term biosafety concerns. Further investigations are expected to result in an improved understanding of the degradative pathways of these polymers within the cells.

In summary, carboxylated amphiphilic polymers with pH-responsive endosomolytic activities demonstrate promising potentials for the intracellular delivery of macromolecules. From a retrospective view, this field has continuously progressed with the application of new synthetic techniques, mechanistic understanding of endocytic trafficking, and better methods for endosomal escape characterization. Up to date, various designs have been made to adapt these polymers for biomedical applications, i.e., the delivery of antigens, genes, and therapeutic peptides. Nevertheless, it is still early to expect clinical translations, due to the lack of biodegradability and long-term biosafety concerns. It would require joint efforts from polymer chemists, biologists, and pharmaceutical scientists to understand how polymers interact with endosomal membranes at the molecular level; how the endosomal escape happens at the cellular level; and finally the delivery in the complicated *in vivo* environment.

## Author Contributions

SW constructed the idea, designed the article, drafted and revised the manuscript. The author has agreed to publish the content of the work.

## Conflict of Interest

The author declares that the research was conducted in the absence of any commercial or financial relationships that could be constructed as a potential conflict of interest.
